# Active aging needs from the perspectives of older adults and geriatric experts: a qualitative study

**DOI:** 10.3389/fpubh.2023.1121761

**Published:** 2023-06-15

**Authors:** Shahla Ayoubi-Mahani, Maryam Eghbali-Babadi, Ziba Farajzadegan, Mahrokh Keshvari, Jamileh Farokhzadian

**Affiliations:** ^1^School of Nursing and Midwifery, Isfahan University of Medical Sciences, Isfahan, Iran; ^2^Nursing and Midwifery Care Research Center, School of Nursing and Midwifery, Isfahan University of Medical Sciences, Isfahan, Iran; ^3^Medicine Faculty, Medical Sciences of Isfahan University, Isfahan, Iran; ^4^Nursing Research Center, Kerman University of Medical Science, Kerman, Iran

**Keywords:** active aging, needs, older adults, geriatric experts, qualitative study

## Abstract

**Introduction:**

With an increasing rate of population aging and its consequences, preparation for active aging based on older adults' needs is an unavoidable priority. Active aging needs must be identified to help strategic planning for older adults' health and wellbeing. This study aimed to explore the active aging needs from the perspectives of older adults and geriatric experts.

**Methods:**

This exploratory-descriptive qualitative study was conducted in four provinces with the oldest populations in Iran. Semi-structured and focus group interviews were conducted with 41 participants (20 older adults and 21 geriatric experts), who were chosen through purposive and snowball sampling. Data were analyzed using the conventional content analysis.

**Results:**

This study identified three themes and thirteen categories from the data: (1) basic individual needs with three categories of physiological, psycho-emotional, and spiritual needs; and (2) managerial needs with seven categories of political-legal, socio-economic, and cultural-spiritual infrastructures, academic strategies, an age-friendly environment; technological services, and provision of specialized services and daycare for older adults, and (3) educational needs with three categories of training self-care and self-efficacy, empowering the health care workers; and empowering the family.

**Conclusion:**

The results revealed personal, managerial, and educational needs for active aging and could assist policymakers and geriatric experts to promote and meet active aging needs successfully.

## Introduction

Population aging is one of the topmost global successes, a reason to celebrate medical advancements, and social, economic, political, and health achievements in any society. Aging has attracted the attention of the world community as a significant demographic issue (1). According to the World Health Organization, the number of older adults will reach two billion by 2050, with about 80% of them living in less developed or developing countries (2). In Iran, it is predicted that people over 60 years old will make up 21.7 percent of the Iranian population by 2050 (3).

Aging leads to many individual and collective challenges, including increased number of people at risk of chronic diseases (4, 5), decreased physical activity, psychological and social vulnerability, and the health system difficulty in providing health care services (6). Aging will cause social, economic, medical, and health crises if not managed and prevented timely (7).

Active aging is one strategy to manage the increasing rate of old population and its complication (8) and is defined as the process of optimizing opportunities for health, participation, and security to enhance the quality of life as people age (9). The goal of active aging is to empower older adults to become active participants in their health care; therefore, comprehensive and precise planning is necessary to improve active aging (8).

Success in aging programs requires considering the viewpoints of policymakers and older adults, who are the biggest beneficiaries of active aging projects (10) because insufficient information can make any program ineffective (11). Furthermore, policies and programs for older adults should be based on their needs, preferences, and capabilities (9). A qualitative study is more suitable than a quantitative one to reach in-depth viewpoints and maximum data from the participants (12); individuals are the best source for describing their situations, needs, feelings, and experiences in a cultural context (13).

The concept of active aging has been used as a research direction in population studies and as a public policy tool over the last few decades, particularly since it was adopted by the World Health Organization to strengthen health, participation, security and lifelong learning as pillars of quality of life. The definition of active aging is a complex task as it is linked to different theories, built from multiple contents and their interactions, linked to other conceptual frameworks of aging (healthy, productive, successful and positive aging) and according to scientific strategies and the shaping public policies are implemented (14). Active aging in Iran is defined according to the cultural background and beliefs of the older adults: the process of individual and social existentialism that is manifested and realized through existential functionalism, management of home affairs, primary isolation, social participation, physical dynamic and vision dynamic-based learning. Therefore, the concept of active aging has both subjective and objective behavioral dimensions (15).

Active aging in society is defined with its indicators in Asian countries, including employment, social participation, independent living and active environment. The difference in societies is dependent on the active aging capacity of the older population in societies and the provision of age-friendly environments. To measure these indicators in Asian societies, the Asian active aging index (ASIAN AAI) is used to quantify the extent to which older adults enjoy active aging and can determine their potentials (16). Active aging programs are implemented in different countries as follows: a training program on active aging in Portugal (17), the active aging program in nursing homes in Spain (18), Green Care program in Italy (19), A national action plan for active and healthy aging in Scotland (20), Vital Aging program in Mexico (21), intergenerational programs to promote active aging in the UK (22), “I am active” in Mexico (23), Opportunities for the Elderly Project (OEP), Elder Academy Scheme and Neighborhood Active Aging Project in Hong Kong (24), and promoting active aging through university programs in Spain (25).

According to the perspective of active aging, aging is a lifelong approach that starts from the beginning of life and its determinants (needs) include health services, socioeconomic factors, physical environment (place of residence and living conditions), the social environment (formal and informal support networks) in the cultural context of people according to gender, race and ethnicity and other social determinants of health such as access to education, employment and health care (26).

The specific needs of older adults must be met to promote active aging, including physical, psychological, social, environmental, care and health-related needs (27, 28). Ashokkumar et al. (29) indicated that older adults had unmet needs such as adaptation to the living environment, hearing and vision problems, income, daily activities, physical diseases, close association, and self-care, while their therapeutic and medicinal needs were met by the caregivers (29). Various studies showed that many of the social, care, and health needs of older adults remained unmet (30, 31). Unmet needs leads to poor quality of life, dissatisfaction with the services provided, and an increase in care costs; therefore, identifying and meeting the needs of older adults can increase their quality of life and satisfaction (32). A systematic review on nine studies suggested limited studies on the needs of older adults in different countries, with physical and social dimensions being the most and least fulfilled needs, respectively, while the physical and social dimension, and psychological and environmental dimension being the most and the least unmet needs. These studies focused on quantitative methods (cross-sectional) and were carried out in Iran, Brazil, Germany, Thailand, Poland, the Netherlands, and England in 2000–2020. The Camberwell Assessment of Need for the Elderly (CANE) was used to assess met and unmet care needs (27). Qualitative studies can specify people's views and experiences more precisely than quantitative research because they enable participants to express themselves while providing data (12).

Considering the population changes and its consequences, Iran must set priority and plan for active aging based on needs (1, 33), but most studies focused on the health-related needs of older adults (32, 34) and did not address all needs of older adults in the Iranian culture and context (11), and there is no comprehensive qualitative study that examines their needs to achieve active aging from the perspectives of older adults and geriatric experts. Moreover, the literature does not sufficiently address qualitative approaches to identify these needs. More rigorous qualitative studies are essential to assess these needs in active aging programs in different contexts. Therefore, this study aimed to explore the active aging needs from the perspectives of older adults and geriatric experts within the culture and context of Iran.

## Methods

### Study design and setting

This descriptive-explorative qualitative study was performed with the use of a conventional content analysis. Content analysis is a subjective interpretation of textual data to obtain new knowledge and insights when research and literature are limited in the context of the intended phenomenon (35).

This study focused on the perspectives and experiences of older adults and geriatric experts affiliated with universities of medical sciences. Older adults were selected from Isfahan that is one of the important cities and the cultural capital of Iran with a population of about 5,386,437, with 12.4 percent of the older people living in Isfahan (666,367). Geriatric experts were selected from Gilan, Tehran, Isfahan, and Tabriz, which were among the ten with the oldest populations (mean old age >60) in Iran. The World Health Organization defines older patients if they are 60 years or older in developing countries (3). In Iran, which is a developing country, older adults are 60 years or older.

In addition to the standard definitions, active aging in Iran is defined according to the cultural background and beliefs of the older adults: the process of individual and social existentialism that is manifested and realized through existential functionalism, management of home affairs, primary isolation, social participation, physical dynamic and vision dynamic-based learning. Therefore, the concept of active aging has both subjective and objective behavioral dimensions (15).

### Participants and sampling

Forty-one participants (20 older adults and 21 geriatric experts) were invited to participate in this study by purposive sampling. The snowball sampling method was also used to reach more samples: the participants could suggest other participants who met the inclusion criteria and were willing to participate. Maximum variation sampling was used to obtain rich information. The inclusion criteria of the participants are shown in Table 1.

**Table 1:** Participants' inclusion criteria.

**Older adults**	**Geriatric experts**
-Over 60 years of age -Ability to convey concepts and participate in the interview -Not having any known Alzheimer's, hearing, or speech problems -Not having physical disabilities in self-care -Not having known mental disabilities -No hospitalization -Willingness to participate in the research	-Geriatric health faculty -Geriatric lecturer Nursing Faculty -Heads of health departments -Nursing home nurse -Psychiatrist -Social medicine -Social worker -Willingness to participate in research -Having at least 2 years of experience in the care and treatment of older adults -Being employed in governmental organizations in relation to society

### Data collection

Data were gathered through guiding questions used in 29 semi-structured and two focus group interviews from February 5 to July 5, 2022 (Table 2). The guiding questions were designed with the consensus of the research team according to the purpose of the study, which was to explore the active aging needs from the perspectives of older adults and geriatric experts. The questions asked in semi-structured interviews and focus group discussions were similar.

**Table 2:** Guiding questions.

**Older adults**	-Can you describe your experiences of being active in old age; what do you do to stay active? -According to your own experiences, what actions and factors encourage you to be more active? -In your experience, what are the needs of old age for you to be able to be an active older adult? -What do you expect from yourself, your family, society, or healthcare workers to have an active aging?
**Geriatric experts**	-What experiences do you have about active aging in older adults? -According to your experiences, what is needed in the aging care system to guide older adults toward active aging? -In your opinion, what do our older adults need to live an active life?
**Probing questions**	Why How Explain more Give an example.

Two focus group interviews lasted 60–90 min and were conducted in person, one with six older people and the other with six geriatric experts; in each focus group interview, one researcher, the interviewer, asked questions to create a friendly environment and encouraged all the subjects to talk about their experiences, while the second researcher took notes. The researchers paid attention to the participants' facial expressions and hand gestures to ask exploratory questions and achieve the purpose of the study.

In depth semi-structured interviews were conducted by the first researcher, who had experience in geriatric nursing and in-depth interview for qualitative studies. First, she introduced herself, gave a brief description of the study's goals, and scheduled an interview date. The interview was conducted face-to-face, audio recorded, and transcribed in a quiet place preferred by the participants; health protocols and physical distance were observed due to the COVID-19 pandemic. Initially, some questions were asked to create a friendly atmosphere and to prompt responses of the participants, and then the interview questions sought participants' experiences and viewpoints. Some interviews with geriatric experts were conducted virtually on Team Link, where it is possible to transmit audio and video simultaneously, or were conducted either online or over the phone of their choice. The interview lasted from 20 to 45 min. Data collection and analysis were done simultaneously until data saturation was attained. The last four interviews were conducted to ensure data saturation, meaning that data saturation occurred when no new concepts were obtained in the interviews and the data were repeated, so data collection was stopped and no new interviews were conducted. In addition, the researchers clearly explained and answered the older adults' questions about the concept of active aging during the individual and focus group interviews.

### Data analysis

The MAXQDA 2020 and the Lundman and Graneheim method were used inductively (36). Individual and focus group interviews were transcribed verbatim and reviewed several times immediately to gain a thorough understanding of the participant's perspective. Then, hidden meaning units and the primary codes were extracted, similar codes were merged to form the categories and finally the themes were created. Codes and categories were created based on the consensus of four researchers, who discussed, checked, and reviewed all the meaning units, codes, categories, and themes. The data reached saturation after interviews with thirty-seven people, and four more interviews were conducted to confirm the categories.

### Trustworthiness

The data trustworthiness was determined using Lincoln and Guba' criteria, including credibility, confirmability, dependability, and transferability (36). The extracted codes were given to three older adults and three geriatric experts to ensure the credibility of the finding; they checked and confirmed whether the researcher represented their ideas well. All the extracted transcripts, codes, and categories were reviewed and approved by the researchers. Furthermore, the researcher attempted to increase the credibility of the data by conducting in-depth interviews, interacting with the participants, combining data collection methods (interview-focus group), and selecting participants with maximum variation (job status, educational level, gender, age, work experience in older adults' scope, marital status). An Audit Trail was used in this study to ensure dependability, and all research processes and stages were recorded. For data confirmability, the researcher sent several interviews, extracted codes, and categories to two researchers, who were acquainted with qualitative research but did not take part in the study.

### Ethical considerations

The Ethics Committee of Isfahan University of Medical Sciences approved this study (Approval ID: IR.MUI.NUREMA.REC.1400.131). Verbal and written informed consent was obtained from all participants before the study. The study purpose and methodology were explained to the participants, who allowed their voices to be recorded and they were informed that the files would be deleted after the research. Participants were aware that their participation was absolutely voluntary and that they could withdraw from the study at any time. All participants received assurances of confidentiality and anonymity. The time and place of the interviews were determined with the participants' agreement. All study data were entered into a database on a password-protected computer that only the researchers had access to. The participants were given the researcher's phone number so that they could learn about the study's findings if desired.

## Results

Forty-one male and female participants, including 20 older adults and 21 geriatric experts were interviewed. Table 3 shows the demographic characteristics of the participants and Table 4 presents the profile of the participants.

**Table 3:** Participants' characteristics.

**Characteristics**	**Older adults**	**Geriatric experts**
Job status	Retired (n:12) Employed (n:5) Housewife (n:3)	Geriatric Health Faculties (n:3) Geriatric lecturer Nursing Faculties (n:4) Heads of health departments (n:2) Nursing home nurses (n:3) Psychiatrists (n:3) Social medicines (n:3) Social workers (n:3)
Education level	Illiterate (n:2) Middle/high school (n:3) Diploma (n:8) Academic (n:7)	Bachelor's degree (n:4) Master's degree (n:3) Specialty (n:3) PhD (n:11)
Gender	Male (n:8) Female (n:12)	Male (n:11) Female (n:10)
Age (year)	60–65 (n:7) 66–70 (n:4) 71–75 (n:4) 76–80 (n:3) 81–85 (n:2)	30–40 (n:5) 41–50 (n:5) 51–60 (n:11)
Work experience in older adults' scope (year)	__	3–6 (n:3) 7–10 (n:7) 11–14 (n:5) >14 (n:6)
Marital status	Married (n:13) Widowed (n:6) Single (n:1)	Married (n:15) Single (n:6)

**Table 4:** Participants' profile.

**Participants**	**Older adult/geriatric expert**	**Job status**	**Educational level**	**age**	**Gender**	**Work experience with older adults**	**Marital status**
P1	Older adult	Retired	Academic	82	Male	-	Married
P2	Older adult	Employed	Diploma	62	Female	-	Widowed
P3	Geriatric expert	Nursing home nurses	Master's degree	44	Female	9	Married
P4	Older adult	Housewife	Illiterate	67	Female	-	Married
P5	Older adult	Housewife	Illiterate	73	Female	-	Widowed
P6	Older adult	Housewife	Diploma	61	Female	-	Married
P7	Geriatric expert	Geriatric Health Faculties	PhD	59	Male	23	Married
P8	Geriatric expert	Social medicines	PhD	57	Male	17	Married
P9	Geriatric expert	Social workers	Bachelor's degree	42	Female	8	Single
P10	Geriatric expert	Social medicines	PhD	59	Female	25	Married
P11	Geriatric expert	Geriatric lecturer Nursing Faculties	PhD	60	Male	15	Married
P12	Older adult	Retired	Diploma	77	Male	-	Married
P13	Geriatric expert	Geriatric Health Faculties	PhD	58	Male	20	Married
P14	Geriatric expert	Nursing home nurses	Master's degree	45	Male	7	Married
P15	Geriatric expert	Geriatric lecturer Nursing Faculties	PhD	59	Female	16	Married
P16	Older adult	Retired	Middle/high school	65	Male	-	Married
P17	Geriatric expert	Heads of health departments	PhD	49	Female	7	Single
P18	Geriatric expert	Geriatric Health Faculties	PhD	54	Male	12	Married
P19	Geriatric expert	Psychiatrists	Specialty	50	Female	9	Single
P20	Geriatric expert	Social medicines	PhD	54	Male	13	Married
P21	Geriatric expert	Geriatric lecturer Nursing Faculties	PhD	55	Female	11	Married
P22	Older adult	Retired	Academic	73	Male	-	Married
P23	Older adult	Employed	Academic	60	Female	-	Single
P24	Older adult	Retired	Middle/high school	64	Female	-	Widowed
P25	Older adult	Retired	Diploma	76	Female	-	Married
P26	Older adult	Retired	Diploma	84	Female	-	Widowed
P27	Geriatric expert	Social workers	Bachelor's degree	37	Male	6	Married
P28	Geriatric expert	Social workers	Bachelor's degree	33	Female	4	Single
P29	Geriatric expert	Psychiatrists	Master's degree	39	Male	7	Married
P30	Older adult	Retired	Diploma	79	Male	-	Married
P31	Geriatric expert	Heads of health departments	Specialty	56	Male	12	Married
P32	Older adult	Employed	Academic	63	Female	-	Widowed
P33	Older adult	Retired	Diploma	71	Male	-	Married
P34	Geriatric expert	Geriatric lecturer Nursing Faculties	PhD	57	Female	12	Single
P35	Older adult	Employed	Academic	64	Female	-	Married
P36	Older adult	Retired	Academic	67	Female	-	Married
P37	Geriatric expert	Nursing home nurses	Bachelor's degree	32	Female	4	Single
P38	Geriatric expert	Psychiatrists	Specialty	40	Male	8	Married
P39	Older adult	Retired	Diploma	74	Female	-	Widowed
P40	Older adult	Retired	Middle/high school	68	Male	-	Married
P41	Older adult	Employed	Academic	66	Male	-	Married

Data analysis indicated three themes, including basic individual needs, managerial needs and educational needs with thirteen categories. The themes and categories with some exemplary quotes are shown in Table 5. We interviewed with older adults and geriatric experts to reach a shared perception of active aging and included the codes obtained from the perspectives of the older adults and geriatric experts in similar categories.

**Table 5:** Interview excerpts supporting key factors related to themes and their categories.

**Themes**	**Categories**	**Example of interview excerpts**
Basic individual needs	Physiological needs	“I enjoy going to sports classes because exercise and walking increase my activity.”(P1, an older adult) “If I do not eat well, I get lethargic. My wife is very careful with me. I believe that older people should be more mindful of their diets and sleep patterns.”(P5, an older adult) “Most people believe that old age is equal to pain tolerance, but older adults have the right to live the best quality of life until the end of their lives. Therefore, one of the important needs is to relieve their pain.”(P3. a geriatric expert)
	Psycho-emotional needs	“Others think that older people do not need their attention because they have grown up. My children visit me less frequently or are preoccupied when I visit them, I can honestly say that I have observed this disregard.”(P2, an older adult) “Unfortunately, older adults' privacy has been ignored, which harms their mental health, self-esteem, and even their relationships.”(P9, a geriatric expert)
	Spiritual needs	“I pray every morning. I even go to the mosque once every few weeks and sort the books there with the ladies. It is both a good deed and gives me peace.”(P4, an older adult) “Participation in religious ceremonies is one of the spiritual needs that make older people feel active. If you look closely, you will notice that older people always arrive in mosques or shrines before the rest.”(P10, a geriatric expert)
Managerial needs	Political-legal infrastructures	“We do not have legal rules for older adults, for example, when you get on a bus, you may see a sign that says: this place is for older people to sit, but if someone does not comply, older people have no right to protest, while in other countries, that older person can file a complaint.”(P8, a geriatric expert) “To keep their old-age population active, some countries have increased the retirement age according to people's ability. For example, France has adopted 35 years instead of 30.” (P11, a geriatric expert)
	Socio-economic infrastructures	“In some European countries, older adults are under insurance coverage, meaning that they no longer have to worry about how to get back their money if they become sick, but older adults in our country, who are unable to pay for their treatment are not covered in the healthcare system, so they slowly get worn out.”(P15, a geriatric expert) “I am retired, but I would like to work. The essence of a human being is work, but unfortunately, it is impossible. I neither have any capital to start a business independently nor does any place accept me because of my age.”(P6, an older adult) “Older people require spending some times with others. For example, if we establish associations or cultural centers where retired older people can gather, converse, and participate in whatever they can do, we will help keep them active.”(P13, a geriatric expert)
	Cultural-spiritual infrastructures	“In our country, people think that when older adults become retired, they must just stay at home and visit others, and when they want to start a new activity, others tease and force them to stay at home. Retired older adults in other countries start traveling around the world. Culture has infused them with the idea that now is the time to focus on yourselves and your desires.”(P7, a geriatric expert) “It has been 10 years since my husband died. My children are married, and I am very alone and would love to have a companion, but I have never spoken to anyone because I am worried about what people might say behind my back or how my children might react.”(P12, an older adult)
	Academic strategies	“Education and counseling for older adults can be provided in the form of third-generation universities, and older adults can learn how to provide self-care and care for others before retiring.”(P14, a geriatric expert) “We should encourage academics to focus more on aging research, challenges, and solutions. Universities and research departments should increase grants for such research.”(P15, a geriatric expert)
	an age-friendly environment	“I cannot use the bus for commuting or shopping because the stairs are so high that I am afraid of falling. A few years ago, one of my friends fell down the stairs and broke her leg.”(P16, an older adult) “If the older adults' homes become age-friendly environments, we can provide the required ground for them to be active. For example, older people with urinary frequency take medicine and become confused. If the toilet ceramics are slippery, they will slip and break their legs when they wake up in the middle of the night to go to the toilet. This factor turns an active person into a dependent one.”(P19, a geriatric expert)
	Technological services	“Once radio and television were the only media we had, but now smart phones and Internet have replaced them. I only know how to call with my smart phone, but my 5-year-old grandson knows about all the apps. I am interested in learning how to make a video call.”(P23, an older adult) “We have all seen an older adult standing in front of an ATM, waiting for someone to withdraw money for him. We are living in the digital age, so we have to train older adults to keep them active.”(P27, a geriatric expert)
	Specialized services and daycare	“Given the disabilities and transportation problems for older adults, we must provide facilities to provide some or all of their care at home. For example, we should support home care programs for older adults with disability, who have no one to care for them, or even those who prefer to be cared for at home.”(P34, a geriatric expert) “At the moment, older adults rarely visit health centers. Follow-up and encouragement to visit can be effective.”(P21, a geriatric expert)
Educational needs	Training self-care and self-efficacy	“They should be trained how to take their medications in different ways, which can make them less dependent on others.”(P18, geriatric expert) “Older people make an association between their mental and physical problems; they set priority on their physical problems and ignore their mental problems, while some physical problems may have psychological roots.”(P38, a geriatric expert)
	Empowering health care workers	“We emphasize the education of older adults and health care workers. For example, consider someone who wants to examine the oral health of older people. She/he must have received training in this field; otherwise, she/he may not detect oral problems and refer the person.”(P17, a geriatric expert) “I went to a health center a few days ago where I had to test for my blood sugar. They inserted the needle through my finger improperly. I protested them, but they said: Sir, an old man like you should tolerate pain!”(P30, an older adult)
	Empowering the family	“I want my children to understand me and do not tell me that my words belong to the past when I say something, ask me more about how I am doing, take me on trips with them just as when we took them with us everywhere in their childhood “(P25, an older adult) “Families with older adults should know how to support them and should not take responsibility away from them. For example, we feed them ourselves, or if they want to bring some tea, we tell them 'Oh, please sit down. This causes older adults to gradually lose their abilities and become inactive.”(P31, a geriatric expert)

### Theme 1: basic individual needs

The participants' experiences of basic individual needs were categorized into three categories, including physiological, psycho-emotional, and spiritual needs.

#### Physiological needs

The participants emphasized physiological needs to perform activities. These needs included observing personal hygiene, eating a healthy diet and getting enough sleep, exercising, participating in regular physical activities, relieving pain, and controlling weight.

#### Psycho-emotional needs

Psycho-emotional needs in active aging included the desire for love, attention, endearment, and kindness. Respecting for older people in the family and society, strengthening social ties, and respecting privacy helped them to be active. Other needs included the ability to share lived experiences with others and the freedom to choose and act.

#### Spiritual needs

Older adults were interested in attending religious rituals. They emphasized the role of prayer and supplication to reach inner peace. Some of them also believed that providing a peaceful environment in life and respecting beliefs were enough to fulfill this need.

### Theme 2: managerial needs

Political-legal, socioeconomic, cultural-spiritual, academic strategies, an age-friendly environment, technological services, and provision of specialized services and daycare for older adults were the seven categories of active aging management needs.

#### Political-legal infrastructures

The political-legal infrastructure requires modeling prosperous aging in the world, creating rules specific to older adults, and drawing the attention of policymakers to the phenomenon of aging. The participants emphasized the importance of policymakers' awareness of the characteristics of an age-friendly city, and their supports and attentions to the special requirements of older adults. They also believed that subordinate institutions should implement announced policies, authorize officials in the field of old age, and allow older adults to continue working during their retirement in case of being able to work.

#### Socio-economic infrastructures

The participants pointed out high treatment costs, demanded special financial, and social facilities and insurance, and wanted their social dignity to be kept. They claimed that a part-time job based on their competence and capability could be effective in keeping them active. They also demanded cultural centers, retirement associations, and social groups to share their experiences with youths, as well as low-cost activities for their leisure time, so that they could be accepted in the community and eliminate their isolation.

#### Cultural-spiritual infrastructures

According to the participants' experiences, the involvement of older adults in social activities, attention to beliefs and traditions in the planning, reduction of generation gap, and group trips must be encouraged; they wanted the end of ageism and the taboo of remarriage and required culturally appropriate, fair care and gender compatibility.

#### Academic strategies

The participants also considered effective academic strategies in achieving active aging, including training geriatric experts, providing research grants for geriatric research, establishing geriatric clinics, and facilitating educational degrees for older adults. They also believed that using the experiences of retired professors could reduce generation gap and improve the abilities of professors and students.

#### An age-friendly environment

This category aimed to keep older adults active at home and in the community and included the adaptation of public places to the presence of older people, change of the home environment to the needs of older adults, the adaptation of public transportation to old age, sports centers suitable for older adults.

#### Technological services

This category included provision of technological services for older adults: encouraging older adults to use new technologies (SMS/video call/sending photos/ATM), setting up a telephone consultation line, creating websites, and allocating them virtual groups related to health and counseling.

#### Specialized services and daycare

Participants requested employment of knowledgeable workforce in the field of geriatrics and assignment of a specific position for geriatric nurses. The geriatric experts and older people all agreed that holistic home care was important for periodic evaluations of older adults. They focused on prevention and screening instead of treatment of older people; follow-up of older adults, easy access to treatment or drugs, and coordination of services in referral were significant in this regard. Older adults needed specialized counseling and care services and rehabilitation centers based on the severity of their dependence. Encouraging older adults to visit the centers, informing them about the available services, and providing daycare services were other necessary needs.

### Theme 3: educational needs

This theme consists of three categories: training self-care and self-efficacy, empowering healthcare workers, and empowering the family.

#### Training self-care and self-efficacy

Knowledge of the aging process and natural signs of aging, symptoms and complications of the disease, and prescription drugs were among the needs mentioned by the participants because they were unaware of the advantages of treatment adherence. Familiarity with the signs of mental disorders and prompt referral can aid in the early detection of mental problems. They also wanted to know how to make an emergency call in emergencies, become familiar with organizations supporting older adults, and understand social laws. They were interested in participating social activities, communicating and interacting with different age groups, and doing memory-enhancing activities. Many older people should be encouraged to spend money on themselves.

#### Empowering the healthcare workers

Participants demanded up-to-date information on their diseases, how to become happy in the event of sickness, how to behave with other older people, as well as understandable and clear answers to their questions. They also focused on in-service training for healthcare professionals regarding the proper screening and assessment of health problems, the importance of respecting their rights at health centers, including a VIP appointment time for older adults who are unable to wait for a long time during the consultation.

#### Empowering the family

Participants believed that their families had to be aware of the problems associated with older adults and how to treat them, allow older adults to make decisions independently and give them some responsibilities to maintain their self-confidence and independence. Families should accompany older adults on health problems and support them if they require money for their financial affairs. Family members should maintain older people's right to live independently, identify their abilities, encourage them to travel with them, and manage family relationships.

## Discussion

The current study aimed to explore the active aging needs from the perspectives of older adults and geriatric experts in Iran. The results of this study were classified into three themes: basic individual needs, managerial needs, and educational needs.

The basic individual needs were physiological, psycho-emotional, and spiritual needs in this study. Older adults experience different physiological needs depending on their age, ability level, and gender (37). According to the literature, most needs of older adults are interdependent (38), so improving physiological needs such as doing regular physical activities and exercise, eating a healthy diet, and sleeping sufficiently all have mutual impacts (39). Physiological needs should be considered when caring for older adults, including access to a balanced diet, a suitable living environment, adequate sleep, and appropriate activity (40). One study emphasized the fulfillment of physiological needs of the older adults (41). Lazarus et al. suggested that regular aerobic exercise met older people's physiological needs and improved their quality of life (42).

The current study found that fulfilled psycho-emotional needs increased motivation of older adults to be active. Several studies showed that meeting the basic psychological needs of older adults could predict their level of wellbeing such as increased compassion and kindness (43), emotional relationships, life satisfaction, mental strength, and vitality (44, 45). Older adults' quality of life and health level can be improved by meeting their basic psychological needs and increasing their motivations (44). Researchers reported numerous studies on the significance of the physical needs of older adults (46–48), which raised public awareness in society, but less attention has been paid to the psychological needs of older adults particularly when considering the role of these needs in achieving active aging (49).

Meeting the spiritual needs of older adults was an important factor in achieving active aging and their beliefs were essential in adapting to the challenges of aging (50); aging increases people's religious beliefs and desire to participate in spiritual rituals (51). According to the literature, the presence and involvement of older people in religious ceremonies and activities can reduce their stress from aging consequences (50), improve their life satisfaction (52), enhance their motivation to create and strengthen self-care behaviors, and subsequently improve their level of health and active aging (53). As a result, spiritual needs should be considered in active aging planning according to the religious context of Iran because spiritual health ensures the integrity of a person characterized by stability in life, peace, tranquility, harmony, and feeling of closeness to God, self, society, and environment.

The managerial needs in this study included political-legal, socio-economic, cultural-spiritual infrastructures, academic strategies, an age-friendly environment, technological services, specialized services, and daycare for older adults. The results of this research emphasize that some acts should be legislated for the rights of older adults as a vulnerable stratum. The government must provide supervisory authorities to deal with the complaints of older people, establish a telephone line as a communication bridge between older adults and the judicial and executive officers to report older adults' rights violations, implement educational programs at the departmental level to honor the older customers and employees, and facilitate applicable administrative processes to protect the rights of older adults (54). Gholipour et al. (55) also emphasized that the government should provide older adults with services to improve the quality of active aging services in a long time. Iran, like other countries, will face an increase in the population of older adults in the coming decades (55).

The study findings showed that the most important point was to create economic security for older adults through employment, the provision of living expenses and health insurance, and free care after retirement (56); many studies focused on employment in active aging programs and policies (57, 58). Therefore, more social support for older adults improves the level of social capital of individuals and related organizations (59). One of the most significant prolonged challenges will be the socioeconomic effects of old age. In Iran, some local activities are entrusted to older adults while involving them in the boards of directors, which will lead to their activity and participation in society and, as a result, the promotion of active aging (60).

Many studies considered older adults' spirituality and spiritual therapy to improve their quality of life and spiritual health, and reduce their anxiety and depression (61, 62). According to the findings, spirituality has a significant impact on one's overall health; thus, religion and spirituality are considered important sources of adaptation to stressful life events (63). As a result, it is imperative to note the beliefs, creeds, and spirituality of older people to promote active aging. One of the critical issues is the presence of a companion and spouse in older people' lives to actively participate in cultural-spiritual activities (64). This reduces the challenges of aging, increases happiness and motivation, and improves the level of physical, mental, and emotional wellbeing (65). Although older adults' remarriage faces many cultural and social obstacles and taboos, particularly in Iran (64), it is recommended to recognize and solve the existing obstacles because it is important in providing active aging and increasing the welfare of older adults. The spiritual viewpoint is only considered a tool in the world to treat disease, but it has a practical definition in Iranian society and culture, which can improve older adults' welfare.

With the corporeal, physical, psychological, and social changes associated with aging, it is necessary to modify and secure the environment for these people (66), so that adaptation to the environment around them is one of the practical components of active aging and leads to a better quality of life in older adults (57). Falling and its complications are one of the major problems for older people because of inadequate safety measures in physical space at home and in society (67), which can lead to mental injuries and worsen their anxiety, fear, social isolation, and, eventually, depression (68), so older people must change their living environments. However, the proportion between the physical conditions of older adults and the environment has not improved in Asian countries (69), so their homes and social spaces must match with each other (66). It is recommended that urban construction engineers use the research findings to provide the required infrastructure and fulfill the needs of older adults. The physical environment is unsafe when caring for older patients, so it is necessary to pay attention to the important role of physical safety in the care centers for the older adults (70).

Providing technological services for older adults was another category of managerial need in the present study. Learning digital technologies is one of the necessities of modern life and plays an effective role in meeting the daily needs of older people, so it is essential to train older people enough information in this field (71). Studies demonstrate that older adults' participation in social media is directly related to their physical and mental health because they remain connected to society, which is beneficial for promoting active aging (72, 73). The rate of acceptance of technology among older adults is noteworthy, but one of the most significant barriers is the difficulty in using some of these tools. Older adults can benefit from the advantages of technologies and be more active on the condition that the offered tools and programs are easy to use for them (74). Thus, older adults should be the target group of technology programmers and designers when designing and creating technologies to improve their abilities while using these tools (75).

Some caregivers provided specialized services and daycare for older adults, indicating that older adults required specialized advice and care services based on the severity of their dependence. Caregivers provide three types of daily services based on the needs of older adults: social services (meals, recreation, and other health-related activities); medical and health services (social activities and medical services), and professional measures (specialized services to older adults) (76). Aseyedali et al. (77) indicated that community-oriented programs, including daycare centers that focused on expanding family, helped older adults prevent their isolation and social exclusion (77). According to the literature, daycare services improve routine activities, life satisfaction, dependency levels, and activity levels in older adults (78), but families are not aware of such centers. Moreover, programs relating to the provision of daycare services may become unstable and reduce access to needed services as a result of a lack of support from policy-making organizations. Most of the integrated geriatric care is directed toward sick older adults, while the care and education of the healthy older people are neglected (79), so this issue should be addressed because it has the potential to reduce the societal costs and diseases while also improving the quality and satisfaction of life for older adults (80).

The educational needs were for three groups: older adults, health care workers, and family. Information about the levels of self-care and self-efficacy in older adults is one of the most important needs and indicators of their independence. Older people, like other groups, need access to information to remain active in society, make informed decisions, maintain and improve health levels, make the right choices, and have an active and effective life (81). Ong-Artborirak and Seangpraw (82) demonstrated that learning self-care and self-efficacy behaviors were critical aspects of a healthy lifestyle because people with high self-care behaviors prioritized their health care (82). Similar to the present findings, a study emphasized the role of empowerment programs in promoting self-efficacy in older adults' self-care behaviors (83). Therefore, older adults must learn self-care and self-efficacy skills to match themselves with old-age conditions and active aging (84).

Studies showed that nurses, who completed continuous education in geriatric nursing care, were more capable of providing care for older adults and had a more positive attitude toward their colleagues, had higher empathy skills, moral sensitivity (85), and communication skills (86). Salar and Ghaljaei (87) supported the current research findings and found that codified educational programs should be included in the agenda of health care workers (87). Many health centers have a passive approach to healthy older people due to the high workload and lack of trained personnel (88). Therefore, given Iran's growing older adult population and the importance of training and empowering staff providing services to older adults, detailed educational planning is required to certify health care workers by planners and nurse managers to achieve active aging (6).

Informing family members to take care of older adults and adapt to the challenges of aging at home can significantly improve older adults' health (93). Park et al. also demonstrated that using family-centered care and training family members in geriatric care and related diseases reduced anxiety, stress, and depression in older people and their families while increasing their proper communication with health care providers (89). Since the family is the most essential pillar of society and is responsible for providing correct and appropriate health care to the patient, the family needs an accurate understanding of the disease when caring for older adults. The family can provide the necessary knowledge, skills, and support to maintain the quality of home care for older adults (90). According to the findings of this research and Ris et al.'s study (91), health officials in health centers should hold training workshops with focus on family empowerment in active aging (91).

The study findings can be used in planning related to the older adults in Iran, including strategic planning. The researchers believe that any active aging program should be based on three foundations: (1) older people and development (participation), which focuses on social needs in order to adapt policies to it, and institutions consider the growth of the older population as an active force, (2) promotion of health and welfare with focus on the need and policies that promote health from childhood to the whole life so that they have a healthy life in old age, (3) insurance of the ability and environmental support (security) that promotes family and community-based policies and provides safe aging (92). According to the study participants, the dynamics of aging and active aging are affected by many interrelated factors such as individual, managerial and educational factors. Today, due to the growing aging population, a special attention should be paid to the phenomenon of aging with special needs. Governments and policymakers need to provide relevant infrastructure and programs and allocate sufficient resources for this purpose. The findings indicated sufficient awareness of the needs and tried to better understand the experiences of older adults and how they became more active in this period. Considering the importance of old age and the need to progress active aging in Iran with regard to specific cultural and religious values, providing special services (federal planning, community planning and family arrangement with environmental support) to this group can help to create suitable conditions for improving the active lifestyle of the older adults.

### Strengths and limitations

The most important strength of the present study is that it pays attention to all aspects of older adults' needs and extracts the areas of achieving active aging within the culture and structure of the present society in five selected cities. Moreover, this study explored active aging needs from the perspectives and experiences of older adults and geriatric experts. Understanding of participants' practical experiences, main concerns, cultural contexts, situations, feelings, and needs can help policymakers and geriatric experts identify and determine specific topics in active aging and enhance the effectiveness of the programs.

Given the limitations of the present study, we explored active aging from the perspectives and experiences of older adults and geriatric experts in Iran, but they may not fully represent all older adults' needs in other contexts and communities because of cultural differences and circumstances. We only examined older adults' perspectives and experiences but did not examine the rate of their fulfilled needs, so further mixed methods studies are required in this regard. On the other hand, it is advised to investigate the perspectives of older adults' family members to better meet their needs. In this study, older adults with physical and mental disabilities were excluded due to their dependence on others and different needs to be active compared to other older people, and it is suggested that a separate study investigates the experiences of these older people with special needs.

### Conclusion

A significant portion of active aging needs is dependent on accurate information and data from the population for whom planning is being done. This issue is much more critical and delicate in developing countries due to the lack of data and information. The results of the present study showed the active aging needs in various personal, managerial, and educational aspects, which were determined from the perspectives of older adults and geriatric experts. Considering the increase in the older adults' population in many societies as well as their vulnerability, which necessitates special services and attention, it is recommended that policymakers prioritize the needs of this vulnerable group to achieve active aging in society, and national and urban planners focus on meeting health and welfare needs of older adults. It is suggested to identify how older adults benefit from the services, to prepare expert staff to provide services to the older adults in these centers, to increase the healthcare insurance coverage, to allocate the resources and expand the number of these specialized centers and to consider factors such as availability, cost-effectiveness and integrity in providing services, so that with all-around support, it not only facilitates adaptation to the specific limitations of old age but also leads to the transition of old age to active age and increases their quality of life.

## Data availability statement

The raw data supporting the conclusions of this article will be made available by the authors, without undue reservation.

## Ethics statement

The studies involving human participants were reviewed and approved by the Ethics Committee of Isfahan University of Medical Sciences approved this study (Approval ID: IR.MUI.NUREMA.REC.1400.131). Written informed consent was obtained from the individual(s) and/or minor(s)' legal guardian/next of kin for the publication of any potentially identifiable images or data included in this article. The patients/participants provided their written informed consent to participate in this study.

## Author contributions

SA-M was responsible for data collection and completed a draft report of the findings. SA-M, JF, and ME-B drafted the manuscript. All authors contributed to the revisions of the manuscript, read, and approved the final version of the manuscript for submission, are accountable for all aspects of the accuracy and integrity of the manuscript following ICMJE criteria, and contributed to the study conception, design, analysis, and interpretation of the data.
